# *In situ* Tip-Recordings Found No Evidence for an Orco-Based Ionotropic Mechanism of Pheromone-Transduction in *Manduca sexta*

**DOI:** 10.1371/journal.pone.0062648

**Published:** 2013-05-03

**Authors:** Andreas Nolte, Nico W. Funk, Latha Mukunda, Petra Gawalek, Achim Werckenthin, Bill S. Hansson, Dieter Wicher, Monika Stengl

**Affiliations:** 1 Department of Animal Physiology, University of Kassel, Kassel, Germany; 2 Department Evolutionary Neuroethology, Max Planck Institute for Chemical Ecology, Jena, Germany; University of Arizona, United States of America

## Abstract

The mechanisms of insect odor transduction are still controversial. Insect odorant receptors (ORs) are 7TM receptors with inverted membrane topology. They colocalize with a conserved coreceptor (Orco) with chaperone and ion channel function. Some studies suggest that insects employ exclusively ionotropic odor transduction via OR-Orco heteromers. Other studies provide evidence for different metabotropic odor transduction cascades, which employ second messenger-gated ion channel families for odor transduction. The hawkmoth *Manduca sexta* is an established model organism for studies of insect olfaction, also due to the availability of the hawkmoth-specific pheromone blend with its main component bombykal. Previous patch-clamp studies on primary cell cultures of *M. sexta* olfactory receptor neurons provided evidence for a pheromone-dependent activation of a phospholipase Cβ. Pheromone application elicited a sequence of one rapid, apparently IP_3_-dependent, transient and two slower Ca^2+^-dependent inward currents. It remains unknown whether additionally an ionotropic pheromone-transduction mechanism is employed. If indeed an OR-Orco ion channel complex underlies an ionotropic mechanism, then Orco agonist-dependent opening of the OR-Orco channel pore should add up to pheromone-dependent opening of the pore. Here, in tip-recordings from intact pheromone-sensitive sensilla, perfusion with the Orco agonist VUAA1 did not increase pheromone-responses within the first 1000 ms. However, VUAA1 increased spontaneous activity of olfactory receptor neurons Zeitgebertime- and dose-dependently. We conclude that we find no evidence for an Orco-dependent ionotropic pheromone transduction cascade in *M. sexta*. Instead, in *M. sexta* Orco appears to be a slower, second messenger-dependent pacemaker channel which affects kinetics and threshold of pheromone-detection via changes of intracellular Ca^2+^ baseline concentrations.

## Introduction

In insects odorants are detected by olfactory receptors (ORs) which form a large receptor family of seven transmembrane domain (7 TM) proteins [1]–[5]. ORs are expressed in the dendrites of insect olfactory receptor neurons (ORNs) which innervate hair-like sensilla on the antenna [6], [7]. ORs have an inverted membrane topology with an extracellular C-terminus in comparison to conventional G-protein coupled 7TM receptors [8]–[12]. Besides the diverse ORs binding various odor ligands, a highly conserved protein with weak homology to ORs, named Or83b in the fruitfly *Drosophila melanogaster*, is co-expressed in ORNs of different insect species [1], [13]–[23]. In the fruitfly Or83b was suggested to be a coreceptor forming OR-Or83b heteromers [8], [24] and was consequently renamed Orco [25]. Orco is a prerequisite for odor detection since it is a chaperone necessary for the localization of ORs to the ciliated dendrites of ORNs [8], [26]. Additional functions of Orco are still under discussion. It was proposed that OR-Orco complexes constitute ligand-gated receptor ion channels promoting ionotropic odor transduction [27], [28]. Both, OR and Orco, were predicted to contribute to the pore of the odor-gated receptor-ion channel complex [27], [29]–[31]. In contrast, there is evidence for various metabotropic signal transduction cascades in different insect species [32]–[34]. While one study [27] suggested that all insect species employ solely ionotropic odor and pheromone transduction, another study [28] found evidence in *D. melanogaster* for both a less sensitive fast ionotropic pathway as well as a slower, more sensitive metabotropic transduction cascade coupled to adenylyl cyclase. In addition, the *D. melanogaster* Orco itself forms a leaky (spontaneously opening) cation channel activated by cGMP/cAMP, which relies on protein kinase C-dependent phosphorylation [28], [35]. In contrast, patch-clamp studies on primary cell cultures of *M. sexta* ORNs as well as tip-recordings of pheromone-sensitive sensilla in intact moths suggested that moths employ different odor transduction cascades depending on stimulus concentration, behavioral context, and Zeitgebertime (ZT, with ZT 0 defined as the beginning of the light phase; see Materials and Methods) [33]. The main sex-pheromone component of *M. sexta*, bombykal (BAL), elicited a sequence of at least three consecutive pheromone-dependent inward currents, which were also triggered by IP_3_ perfusion of ORNs [33], [36]–[38]. The first, very rapid and transient pheromone-dependent Ca^2+^ inward current, which lasted less than 100 ms, triggered a sequence of Ca^2+^-dependent ion channel openings. While Orco is present in *M. sexta* pheromone-dependent trichoid sensilla [22], [39], it is not known whether additionally an Orco-dependent ionotropic pathway is responsible for the first pheromone-dependent trigger current in *M. sexta* ORNs, which resembled an IP_3_-dependent Ca^2+^ current.

To investigate the function of Orco in pheromone transduction of *M. sexta*, we examined the effect of the Orco agonist VUAA1 [40]. If OR-Orco heteromers form BAL-gated ion channels, co-activation of Orco with VUAA1 during pheromone stimulation would mimic stimulation with higher pheromone doses. Thus, the Orco agonist would increase pheromone responses dose-dependently within the first ∼25 ms (the first 6 action potentials) of the BAL response. In calcium imaging experiments on HEK 293 cells transiently or stably transfected with MsexOrco it was confirmed that VUAA1 is an MsexOrco agonist. However, in contrast to our expectations, VUAA1 perfusion of trichoid sensilla in intact *M. sexta* did not augment pheromone transduction within the first ∼25 ms nor in the first 1000 ms of the pheromone response. Instead, Orco appears to be a spontaneously active ion channel, which affects spontaneous activity day-time-dependently on a slower time scale, possibly via sustained changes in the baseline Ca^2+^ concentration.

## Results

It is not known if Orco is involved in the first rapid pheromone response in *M. sexta*. Consequently, we stimulated Orco *in situ* with its agonist VUAA1 during non-saturating BAL-stimulations [41]. First, we established whether VUAA1 is an MsexOrco agonist. Secondly, we tested whether MsexOrco forms a spontaneously active, Ca^2+^-permeable cation channel as in *D. melanogaster* [27]. Finally, we challenged the hypothesis that insects in general employ solely ionotropic odor transduction [27] in tip-recordings from intact *M. sexta* pheromone specific sensilla. Different time windows of the pheromone response were evaluated separately to distinguish ionotropic or metabotropic signal transduction cascades. Since *M. sexta* responds with different sensitivity to pheromone stimulation in the sleep-wake cycle [42]–[44] the effects of VUAA1 infusion into pheromone-sensitive trichoid sensilla were compared at ZT 1–3 (activity phase) and ZT 9–11 (resting phase).

### MsexOrco Forms a Spontaneously Active Cation Channel which is Activated by VUAA1 in a Heterologous Expression System

**Figure 1:**
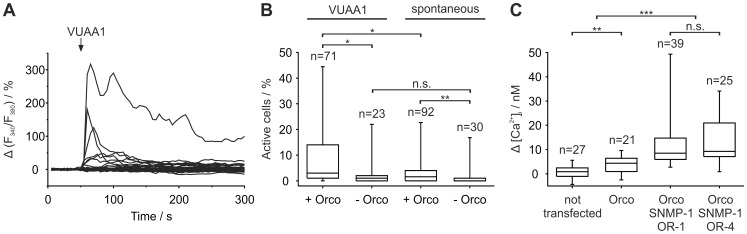
Heterologously expressed MsexOrco is activated by VUAA1 and increases spontaneous activity. Furthermore, MsexOrco appears to interact with MsexORs and/or SNMP-1 in heterologous expression systems. (A) Normalized calcium imaging data of HEK 293 cells transiently transfected with MsexOrco. Data for 100 cells are shown, where each line represents the percentage deviation of the fluorescence ratio (Δ(F_340_/F_380_)) for one cell. After VUAA1 application (100 µl of 100 µM, arrow) eight of 100 cells show an increase in the fluorescence ratio. (B) Percentages of active cells either transiently transfected with MsexOrco (+Orco) or not (-Orco) after VUAA1 application or without application (spontaneous) are compared (n = number of experiments; for each experiment the percentage of active cells was determined). (C) Box plots show the mean increase in the free intracellular Ca^2+^ concentration (Δ[Ca^2+^]_i_) after VUAA1 application. HEK 293 cells were either not transfected or stably transfected with MsexOrco and optionally cotransfected with MsexSNMP-1 and MsexOR-1, or MsexOR-4 (n = number of cells). (B,C) Significant differences are indicated by asterisks (n.s. = not significant; *P<0.05, **P<0.01, ***P<0.001; Mann-Whitney test).

In Ca^2+^ imaging experiments stimulation with 100 µM VUAA1 increased intracellular Ca^2+^ concentrations in HEK 293 cells transiently transfected with MsexOrco (**Fig. 1A**). We confirmed that VUAA1 is an MsexOrco agonist since significantly more MsexOrco transfected cells (median: 3%) showed VUAA1-dependent intracellular Ca^2+^ concentration increases compared to controls not transfected with MsexOrco (median: 1%; **Fig. 1B**). Additionally the percentage of MsexOrco transfected cells showing VUAA1-dependent Ca^2+^ concentration increases was significantly higher than the percentage showing spontaneous Ca^2+^ concentration increases (*P* = 0.024; median: 1.78%). Moreover, MsexOrco transfected cells showed significantly more spontaneous Ca^2+^ concentration increases (*P = *0.003) than non-Orco transfected cells (median: 0%). From these measurements we conclude that MsexOrco forms a leaky Ca^2+^-permeable ion channel, whose open-probability can be increased by VUAA1.

Since the majority of transiently transfected cells did not respond to VUAA1 stimulation, apparently due to sparse membrane insertion of MsexOrco, we obtained a HEK 293 cell line with stably transfected MsexOrco. Similar to the transiently transfected cells less than 5% of the HEK cells responded to VUAA1 stimulation (n = 213). Ca^2+^ concentration increase after stimulation with 100 µM VUAA1 was small but significantly different from responses of non-transfected cells (*P = *0.003, **Fig. 1C**). Cotransfection with MsexSNMP-1 and pheromone receptor candidates MsexOR-1 or MsexOR-4 [39], [45] did not change the percentage of responding cells, but significantly increased the VUAA1-dependent Ca^2+^ concentration increase compared to non-transfected and solely Orco transfected cells (*P*<0.001 for all, **Fig. 1C**). From these measurements we conclude that MsexOrco interacts with MsexORs and/or MsexSNMP-1.

### VUAA1 does not Increase Pheromone Responses in *M.* *sexta*

**Figure 2:**
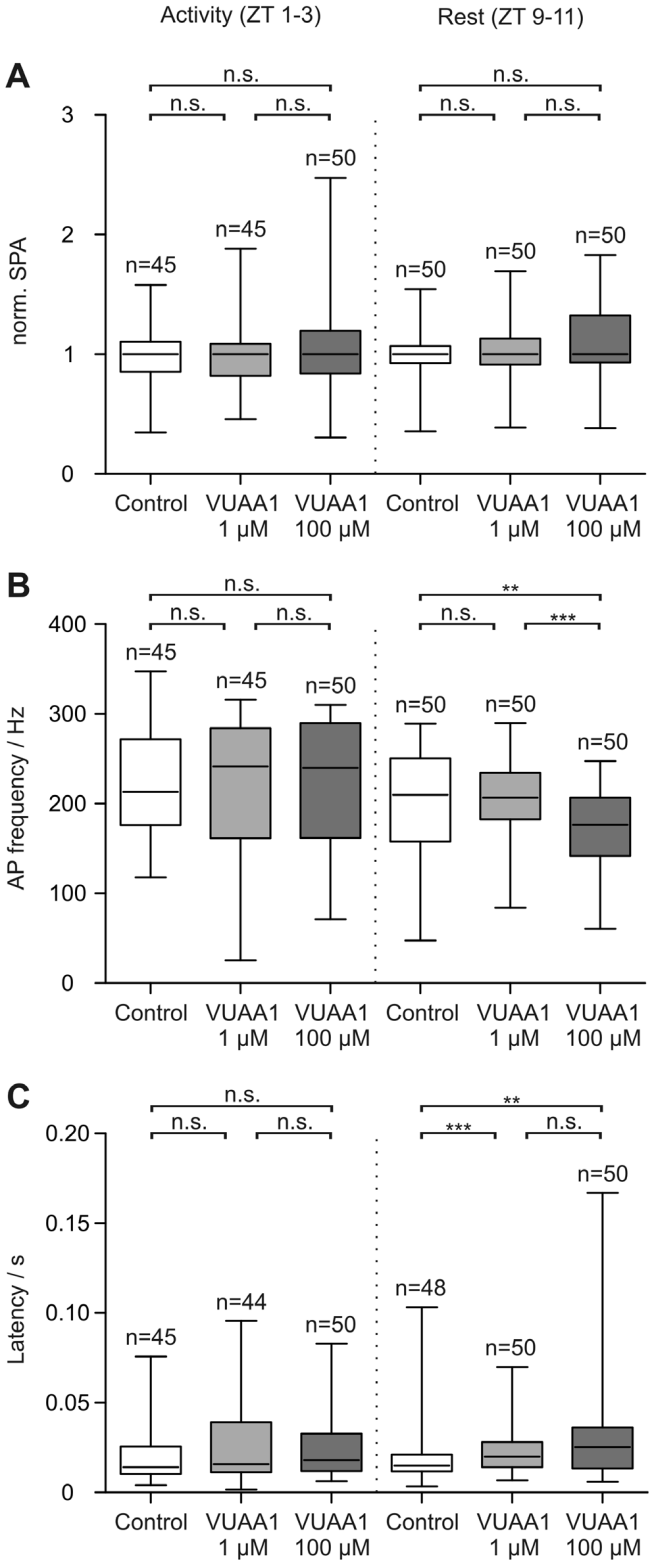
VUAA1-dependent MsexOrco activation does not increase the pheromone-dependent sensillum potential amplitude (SPA) nor the bombykal-dependent action potential (AP) frequency as it would be expected for an Orco-based ionotropic mechanism of pheromone transduction. (A–C) Box plots represent pheromone responses during the first 20 minutes (beginning) of the tip-recordings. (A) The pheromone-dependent SPA was never affected by VUAA1. (B) The pheromone-dependent AP response (first 5 APs) was not affected by VUAA1 during the activity phase, but was decreased at high agonist concentrations at rest. (C) Only at rest the first pheromone-dependent AP was delayed by VUAA1. Significant differences are indicated by asterisks (n.s. = not significant; **P<0.01, ***P<0.001; Mann-Whitney test).

In long-term tip-recordings infusion of VUAA1 did not affect pheromone-dependent sensillum potential amplitudes (SPA), neither during activity nor during resting phase (**Fig. 2A,** **S1;** **Tab. S2,S4**). Thus, Orco appears not to contribute to the rise of the BAL-dependent receptor potential. Also, analysis of the phasic action potential (AP) response did not provide evidence for a BAL-gated OR-Orco-dependent ion channel opening during the first ∼25 ms of the pheromone response. In the beginning of the long-term tip-recordings the BAL-dependent AP frequency was not affected by VUAA1 during the activity phase (**Fig. 2B**). A significant decline only occurred with perfusion of 100 µM VUAA1 at rest. Both control and VUAA1 recordings showed a significant decline in AP frequency over the time course (**Fig. S2A–D; Tab. S2,S4**). This decline was significantly stronger in the presence of 100 µM VUAA1.

The latency of the first BAL-dependent AP remained unchanged in the beginning of the recordings during the activity phase (**Fig. 2C**). However, it was significantly prolonged for VUAA1 at rest (**Fig. S2A–D;Tab. S2,S4**). For both control and VUAA1 recordings the latency increased over the time course. This increase was significantly higher in the presence of 100 µM VUAA1 (**Fig. S2E,F;Tab. S2,S4**).

**Figure 3:**
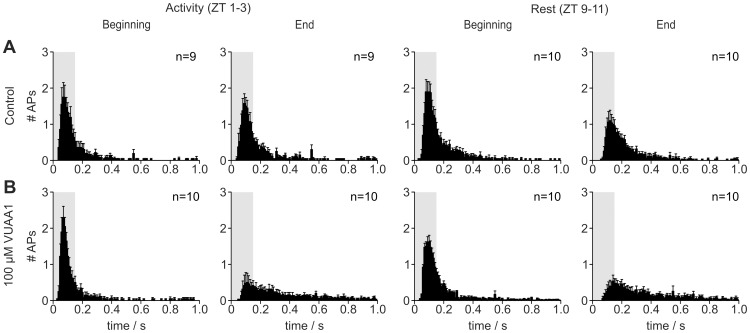
During the first 20 min of each recording VUAA1-dependent MsexOrco activation does not affect the first 150 ms or first 1000 ms of the pheromone response. Rather, MsexOrco-ion channel opening affects bombykal (BAL)-response kinetics at the time scale of minutes, at the last 20 min of the 2 h recording. (A,B) Post stimulus time histograms show the mean number of APs generated within the first 1000 ms after BAL stimulation (binsize = 10 ms). The number of APs within the first 150 ms (shaded area A,B) and the first 1000 ms did not change VUAA1-dependently during the first 20 minutes (beginning) of the recording. At the end of the tip-recordings (last 20 minutes) the pheromone responses shifted to a more tonic response pattern in the presence of 100 µM VUAA1 as compared to the beginning (see also Fig. S3).

To determine further ion channel activation by VUAA1 within the first second of the pheromone response, post stimulus time histograms (PSTHs) were prepared (**Fig. 3**). The number of BAL-dependent APs was analyzed during the first 150 ms and 1000 ms after onset of the BAL-dependent sensillum potential (**Fig. S3, Tab. S2**, **S4**). In the beginning of the recordings neither VUAA1 concentration had any effect on the number of APs generated in the first 150 ms (**Fig. 3A,B** **; Tab S2,S4**). Neither was the number of APs during the first 1000 ms of the activity phase affected by VUAA1 (**Fig. S3C,D;Tab. S2,S4**), while at rest only 1 µM VUAA1 caused a significant decline (**Tab. S2,S4**). Comparison of the distribution (**Fig. 3**) and number of APs (**Fig. S3**) in controls over the course of the recordings indicates that the kinetics of the pheromone response shifted to a more tonic response pattern during rest. In addition, the decrease of the number of APs in the first 150 ms in control experiments indicated an increase in threshold at rest (**Fig. S3, Tab. S2,S4**). Application of the Orco agonist further enhanced this shift in kinetics and BAL-sensitivity during the course of the recording. This suggests that Orco activation affected the kinetics as well as the sensitivity of the BAL response on the time scale of minutes rather than milliseconds.

### VUAA1 Increased Spontaneous and Background Activity

**Figure 4:**
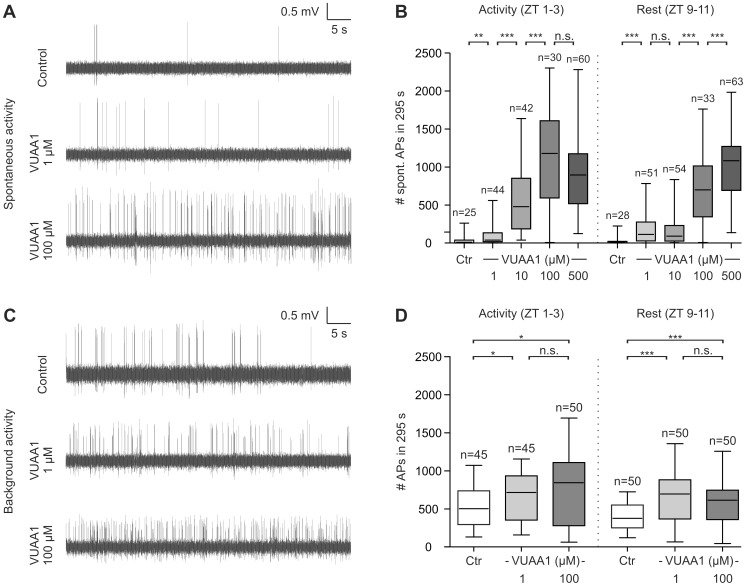
VUAA1- dependent MsexOrco activation increases spontaneous as well as background activity of olfactory receptor neurons (ORNs). Original recordings show spontaneous activity without previous BAL-stimulation (A) and background activity (C) after BAL-stimulation of bombykal- (BAL) sensitive ORNs (larger amplitude). Action potentials of smaller amplitude were generated by the second BAL-insensitive ORN. (B) Spontaneous activity was dose-dependently increased by VUAA1 stimulation, with lower VUAA1 concentrations required for saturation in the activity phase. (D) Lower VUAA1 concentrations increased the background activity already maximally during the first 20 min (beginning) of the recordings and more strongly than the spontaneous activity. Furthermore, MsexOrco appeared to express Zeitgebertime-dependent changes. Significant differences are indicated by asterisks (n.s. = not significant; *P<0.05, **P<0.01, ***P<0.001; Mann-Whitney test).

Next we examined whether Orco forms an ion channel involved in modulating spontaneous activity in the absence of pheromone. A significant VUAA1 dose-dependent increase in spontaneous activity without previous pheromone stimulation could be observed during activity and resting phase, with higher sensitivity to VUAA1 during activity phase (**Fig. 4B;** **Tab. S3,S4**). Also, the background activity between two pheromone stimulations was examined. Application of VUAA1 significantly increased background activity (**Fig. 4C,D;** **Tab. S2,S4**). However, no dose-dependent effect was found. While long-term control recordings showed a continuous decline over the time course, the decline was counteracted in 100 µM VUAA1 recordings during activity and resting phase (**Fig. S4**). Background activity was significantly higher than spontaneous activity in control and 1 µM VUAA1 recordings (*P*<0.001 for all). However, during the activity phase spontaneous activity was significantly higher than background activity under the influence of 100 µM VUAA1 (*P = *0.006).

**Figure 5:**
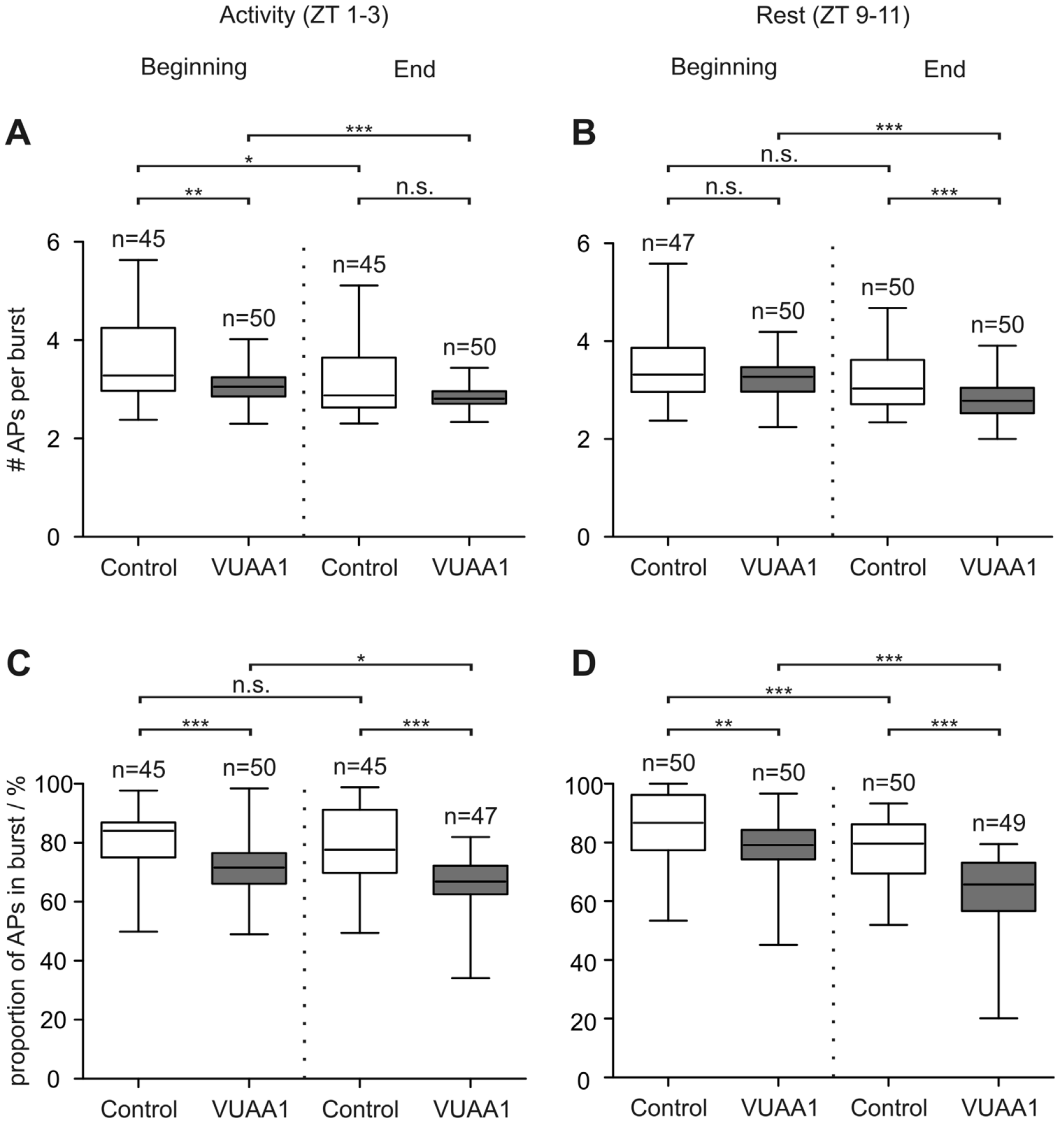
VUAA1-dependent MsexOrco activation affects bursting pattern of background activity. Comparison of the beginning (0–20 min) and end (100–120 min) of long term recordings showed a decrease of the number of action potentials (APs) per burst (**A,B**) as well as the percentage of APs in bursts (**C,D**) in the presence of 100 µM VUAA1. Furthermore, MsexOrco-dependent effects were mostly Zeitgebertime-dependent. Significant differences are indicated by asterisks (n.s. = not significant; *P<0.05, **P<0.01, ***P<0.001; Mann-Whitney test).

Additionally, we analyzed whether VUAA1 affects the bursting pattern in the background activity of the BAL-sensitive ORN. The percentage of APs belonging to bursts and the number of APs per burst were calculated (**Fig. 5**). In the beginning of the recordings both VUAA1 concentrations decreased the number of APs per burst except for 100 µM VUAA1 at rest. The percentage of APs in bursts was always decreased by VUAA1. Addition of 100 µM VUAA1 significantly decreased the number of APs per bursts as well as the percentage of APs in bursts over the time course.

## Discussion

Research in insect olfaction proposed controversial models of odor transduction [27], [28], [32], [33]. Here, with long-term tip-recordings *in situ* from pheromone-sensitive trichoid sensilla of intact *M. sexta* we examined whether MsexOrco-dependent ionotropic pheromone transduction is employed. In heterologous expression systems we confirmed that VUAA1 [40] is an MsexOrco agonist and that MsexOrco forms a spontaneously opening Ca^2+^-permeable cation channel, which appears to interact with co-transfected MsexORs/MsexSNMP-1 [46]. Unexpectedly, with *in situ* studies we found no evidence for the participation of an MsexOR-MsexOrco-dependent ionotropic transduction pathway during the first ∼25, 150, or 1000 ms of the pheromone response during the first 20 min of the tip-recordings. Instead, MsexOrco affects pheromone response kinetics and sensitivity in pheromone- and ZT-dependent manner within the time period of minutes, possibly via its effects on spontaneous activity and pheromone-dependent background activity. We hypothesize that pheromone dependency results from pheromone-dependently activated metabotropic cascades, which changed open-probability and/or conductance of MsexOrco ion channels. We assume that ZT-dependency resulted from different Ca^2+^ baseline levels (which modulate MsexOrco) between rest and activity phase, possibly regulated via a circadian clock in ORNs [33], [47], [48]. Also differences in the pheromone response between beginning and end of long-term tip-recordings are most likely due to changes in Ca^2+^ baseline levels, possibly via the accumulation of DMSO. We suggest that MsexOrco forms a leaky second-messenger-dependent cation channel that controls membrane potential oscillations and intracellular Ca^2+^ baseline levels, and thereby kinetics and threshold of pheromone responses in *M. sexta*.

### VUAA1 Activates MsexOrco

The discovery of different agonists and antagonists of the conserved coreceptor Orco has greatly facilitated the study of Orco function [31], [40], [49]–[54]. Since spontaneous Ca^2+^ signals occurred more frequently in HEK 293 cells expressing MsexOrco (**Fig. 1B**) it can also form functional leaky (spontaneously opening) ion channels as reported for *D. melanogaster* and *Anopheles gambiae* [27], [28], [35], [40]. In addition, 100 µM VUAA1 activated MsexOrco as it does in different other species such as *A. gambiae, D. melanogaster, Culex quinquefasciatus, Harpegnatos saltator*, *Heliothis virescens* and *Ostrinia nubilalis* [31], [40], [49], [50], [52]. The small number of responding cells is very likely due to insufficient membrane insertion of MsexOrco in transiently as well as stably transfected cells. This indicates that in the heterologous vertebrate expression system important components necessary for efficient membrane targeting of MsexOrco are missing. It cannot be decided whether the increase in the VUAA1-dependent current after coexpression with MsexSNMP-1 and MsexOR-1, or MsexOR-4 (**Fig. 1C**) can be attributed to improved membrane insertion of MsexOrco, or to heteromultimerization and formation of ion channels with larger conductance. In summary, we conclude that VUAA1 is a suitable activator of MsexOrco ion channels.

### MsexOrco does not Increase Bombykal Responses

Previous studies showed that Orco itself is not activated by odors in different species such as *D. melanogaster, A gambiae and C. quinquefasciatus* [24], [27], [28], [31], [40], [50], [55] and that VUAA1 binds to Orco directly increasing its ion channel open probability across species [31], [40], [49]–[52]. Therefore it was suggested that VUAA1 is an allosteric activator of possible OR-Orco heteromers, and that VUAA1 addition to the odor-sensitive sensillum should mimic an increase in odor concentration. Thus, VUAA1 and pheromone should act additively and non-competitively in opening the OR-Orco ion channel pore. If indeed MsexOR-MsexOrco heteromers form pheromone-gated ion channels responsible for the first pheromone-dependent inward current, infusion of VUAA1 should increase BAL-dependent receptor potentials and AP responses in *M. sexta*. Unexpectedly, this was not the case. Since neither the BAL-dependent SPA (**Fig. 2A,** **S1**), nor the phasic BAL-dependent AP response during the first ∼25 (**Fig. 2B,** **S2A–D**), 150, and 1000 ms (**Fig. 3,** **S3**) during the first 20 min of the long-term tip-recordings increased VUAA1-dependently, it is very unlikely that MsexOR-MsexOrco heteromers form BAL-gated ion channels in *M. sexta*. This lack of a VUAA1 effect cannot be due to a saturation of the BAL response, since higher BAL concentrations employed previously resulted in dose-dependent increases of the pheromone response [41]–[44]. Since a 100-fold lower VUAA1 concentration did not affect pheromone-dependent AP frequency (**Fig. 2B**) it is very unlikely that VUAA1 failed to activate MsexOR-MsexOrco due to adaptation. Furthermore, because VUAA1 dose-dependently increased the background and spontaneous activity of *M. sexta* ORNs (**Fig. 4** **, S4**) during the first 20 min of the tip-recordings, MsexOrco is sensitive to VUAA1 concentrations employed *in situ* as well as *in vitro* and the agonist successfully reached its target during this time window. Since VUAA1-dependent MsexOrco activation at all concentrations and at all ZTs tested never affected the SPA (**Fig. 2A,** **S1**) over the 2 h recording, MsexOrco does not appear to contribute to the BAL-dependent receptor potential. This assumption is consistent with the observation that Orco activation did not change the number of pheromone-dependent APs in the beginning of the tip-recordings (**Fig. 3,** **S3**). Nevertheless, MsexOrco activation significantly decreased the BAL-dependent phasic AP response during long-term tip-recordings with 100 µM VUAA1 (**Fig. S3**). This observation, together with a significant VUAA1-dependent latency increase (**Fig. 2C**) of the first BAL-dependent AP is reminiscent of cGMP-dependent adaptation observed previously [42]. This could indicate a sustained activation of a spontaneously opening ion channel in ORNs, which increases the intracellular cGMP levels Ca^2+^-dependently and thus shifts the dose-response range of pheromone responsiveness to more adapted response ranges.

### MsexOrco-activation by VUAA1 Affects Background Activity and Spontaneous Activity

As previously reported, Orco is involved in the generation of spontaneous activity in *D. melanogaster* and *A. gambiae in situ* [26], [40], [56], [57]. Moreover, Orco was shown to form a leaky ion channel affecting spontaneous activity in different insect species such as in *D. melanogaster* and *A. gambiae* [27], [28], [35], [40]. The same function could also be confirmed for MsexOrco *in situ* since VUAA1 significantly increased the spontaneous activity of the non-stimulated BAL-sensitive ORNs (**Fig. 4A,B**). Furthermore, the VUAA1-dependent activation of spontaneous activity in ORNs, which never experienced pheromone stimulation before, was significantly different from VUAA1-dependent stimulation of background activity (**Fig. 4**). It is possible that pheromone-dependent metabotropic transduction cascades changed concentrations of intracellular messengers such as Ca^2+^ and cGMP, which then modified the open probability of MsexOrco as reported previously for Orco from *D. melanogaster* [28]. Furthermore, the VUAA1-dependent decrease in the percentage of APs belonging to bursts (**Fig. 5C, D**) correlated with the slowing of the response kinetics, which became more tonic with VUAA1 (**Fig. 3,** **S3**). This direct correlation between bursting behavior and pheromone response kinetics was also observed for octopamine application, which increased the number of APs in spontaneous bursts and sped up pheromone response kinetics as well as increased pheromone sensitivity [44]. Taken together, we believe these data indicate that in *M. sexta*, Orco is a spontaneously opening ion channel, which allows for spontaneous membrane potential oscillations in the absence of pheromone, rendering the cell sensitive to the timing of the input as prerequisite for temporal encoding. Pheromone then might increase the conductance of this possibly circadian clock-controlled Orco-pacemaker channel dose-dependently via pheromone-dependent metabotropic transduction cascades, which change intracellular second messenger levels. Thereby, response kinetics and sensitivity of pheromone responses might be modified Orco- and second messenger-dependently. Current experiments examine whether ZT-dependent differences in VUAA1 effects are due to circadian expression of MsexOrco and MsexOrco-dependent circadian changes of intracellular Ca^2+^ concentrations.

In summary, BAL transduction in *M. sexta* apparently involves a phospholipase Cβ-dependent metabotropic signal transduction cascade without evidence for the involvement of an additional MsexOrco-based ionotropic transduction cascade [33], [36]–[38], [41]. A multitude of different second messenger-gated ion channels, amongst them MsexOrco, regulates the pheromone response range and kinetics ZT- and dose-dependently allowing for gain control and differentiated behavioral responses [33]. More *in situ* studies are necessary to determine whether an Orco-based ionotropic mechanism plays a relevant role in odor transduction *in vivo* in other insects such as *D. melanogaster*.

## Materials and Methods

### Animals and Preparation

All experiments were performed on adult males of the hawkmoth *Manduca sexta* raised in breeding facilities at the University of Kassel as reported previously [43]. Animals were entrained to a 17 h∶7 h light:dark cycle, where Zeitgebertime (ZT) 0 defines the beginning of the light phase and ZT 17 the beginning of the dark phase. Since *Manduca sexta* is a nocturnal insect species, mating behavior, male flight and oviposition occurs in the dark phase [58]. For further information regarding raising conditions and preparatory work see **SI Materials and Methods**.

### Odorant Receptor Expression in Heterologous Systems

The odorant receptors (MsexOR-1, MsexOR-4), MsexOrco ( = MsexOR-2) and sensory neuron membrane protein 1 (MsexSNMP-1) were identified previously [22], [39], [45]. The DNA was cloned into pcDNA3.1(-) expression vectors (Invitrogen) using standard molecular biology methods (**SI Materials and Methods;Tab. S1**). Human embryonic kidney cells (HEK 293, DSMZ) were grown on poly-L-lysine (0.01%, Sigma-Aldrich) coated coverslips. Further culture conditions and transient transfection were described by Wicher et al. [28]. Additionally, HEK 293 cells stably expressing MsexOrco were purchased from cytobox UG (Konstanz, Germany) and grown in cytobox™ HEK select medium containing puromycin. Since the sensory neuron membrane protein 1 (SNMP-1) is coexpressed with pheromone receptors in moth ORNs [59]–[63] and SNMP-1 was shown to be required for pheromone detection in *D. melanogaster* [56], [64] for some experiments, HEK 293 cells were transiently transfected with MsexSNMP-1 and MsexOR-1 or MsexOR-4.

### Calcium Imaging

Calcium imaging experiments were performed on HEK 293 cells using Fura-2 [65] as calcium indicator. Cells were loaded by incubation in culture medium containing 2.5–5 µM of membrane permeable Fura-2 acetoxymethyl esters (Molecular Probes, Invitrogen) for 30–60 min at room temperature. Fura-2 was excited sequentially with wavelengths of 340 and 380 nm using a monochromator (Polychrom V, Till Photonics), coupled to an epifluorescence microscope (Axioskop FS, Zeiss, Jena, Germany) and controlled by an imaging control unit (ICU, Till Photonics). Cells were monitored using a 40× objective (LUMPlanFI/IR 40×/0,80W, Olympus) or a 10× objective (ACHROPLAN 10×/0,30W Ph1, Zeiss). Exposure times varied to achieve sufficient signal to background ratios for both excitation wavelengths. Emission for both excitation wavelengths was detected at 510 nm. Experiments lasted 5 min with a sampling interval of 5 s. VUAA1 (100 µl of 100 µM) was applied via pipette or via rapid solution changer (RSC, Bio-Logic, Claix, France) (**SI Materials and Methods**).

### Tip-recordings

All recordings were performed at room temperature (19–22°C) in the end of the activity phase (ZT 1–3) and during the resting phase (ZT 9–11). A glass electrode filled with hemolymph Ringer was used as indifferent electrode and was inserted in the truncated end of the male’s antenna. The recording electrode filled with sensillum lymph Ringer was slipped over the truncated sensillum [42]. The Orco agonist VUAA1 (1 µM or 100 µM) was applied passively via the sensillum lymph Ringer solution. Long-term tip-recordings lasted 2 h, recordings with 1 µM VUAA1 or spontaneous activity recordings lasted 20 min. Non-saturating, non-adapting pheromone stimulations of 50 ms duration with 1 µg BAL dissolved in 10 µl hexanol on filter paper were performed every 5 min [41], [43]. Neuronal activity of the pheromone sensitive ORN between pheromone stimulations was recorded for 295 s (except the first 5 sec of the pheromone response) and was defined as background activity. Spontaneous activity of non-stimulated ORNs was measured in isolated moths not exposed to pheromone before. Spontaneous activity was recorded for the first 295 s under control conditions and if applicable subsequently from the same sensillum for another 3× 295 s in the presence of VUAA1 (1, 10, 100 or 500 µM). For further details see **SI Materials and Methods**.

### Data Analysis and Statistics

Calcium imaging: Tillvision software (Version 4.5, Till Photonics) was employed to subtract background fluorescence, to define regions of interest (ROIs), and to calculate the ratio of fluorescence resulting from excitation at 340 nm and 380 nm (F_340_/F_380_). Only for the experiments with stably transfected HEK 293 cells the intracellular Ca^2+^ concentration was calculated on the basis of the measured fluorescence intensities as described before [28], [65]. Mean change of the intracellular Ca^2+^ concentration (Δ[Ca^2+^]_i_) was determined from the area under the curve (AUC) over the time courses of the respective cells based on the Ca^2+^ concentration before VUAA1 application using Excel (2007, Microsoft Office). Matlab (Version R2012a, The MathWorks) was used to normalize imaging data and to determine the percentage of active cells (**Materials and Methods SI**).

All tip-recordings were analyzed using Spike2 software (version 7.01, Cambridge Electronic Design). The interval between two pheromone stimuli was divided into a direct stimulus response (5 s) and the following background activity (295 s). For statistics beginning (0–20 minutes) and end (100–120 minutes) of long-term tip-recordings were considered. The following parameters of the direct stimulus response were analyzed: The sensillum potential amplitude (SPA), defined as BAL-dependent negative deflection of the transepithelial potential, was evaluated as measure of the graded receptor potential. For statistical tests the SPA was normalized to the first data point of the recording. Since only the phasic component of the phasic-tonic BAL-response encodes stimulus quantity [41], the first 5 interspike intervals (∼ the first 25 ms time window containing the first 6 APs) of the BAL response were analyzed as parameter for frequency encoding. To investigate temporal encoding the latency between the first AP after BAL stimulation and the onset of the SPA was determined. To examine encoding in the phasic-tonic response pattern the first 150 and 1000 ms after the onset of the BAL-dependent sensillum potential were analyzed and post stimulus time histograms (PSTHs, binwidth = 10 ms) were prepared for the beginning and the end of long-term tip-recordings. Analysis of the background activity was performed as follows: the number of APs was evaluated and the percentage of APs associated with bursts as well as the mean number of APs per burst was analyzed. A burst was defined as two or more APs with maximum interspike intervals of 50 ms.

All statistical tests were performed with Graphpad Prism (Version 5.01, Graphpad Software Inc.; **Tab. S2,S3,S4**. Shapiro-Wilk test was used to test for normality (not shown). Since the majority of data groups did not show a normal distribution all data groups were compared using Mann-Whitney test. The significance level α = 0.05 was used for all tests. Compared data are presented as box plots, showing lower and upper quartile with median (box) and whiskers from minimum to maximum. Figures were generated with Graphpad Prism, Origin (Version 8.6) and Corel Draw (Version X3).

## Supporting Information

Figure S1 **VUAA1 does not increase sensillum potential amplitude (SPA).**
(TIF)

Figure S2 **VUAA1-dependent MsexOrco activation affects the threshold of pheromone-responses during the course of the 2 h-long recording, except during the first 20 min at the activity phase.**
(TIF)

Figure S3 **Orco agonist VUAA1 (100 µM) slows the kinetics of the bombykal (BAL) response.**
(TIF)

Figure S4 **VUAA1 increased background activity over the time course of the 2 h-long recordings.**
(TIF)

Table S1 **Primer sequences.** Coding sequences are shown in capitals. If a restriction site was induced, the respective sequence and appropriate enzyme is indicated with fat letters. Abbreviations: for = forward primer, rev = reward primer.
(DOCX)

Table S2 **Statistics for tip-recordings.** Data groups were compared using Mann-Whitney-test (α = 0.05). Corresponding *P*-values are shown.
(DOCX)

Table S3 **Statistics for tip-recordings: Spontaneous activity.** Data groups were compared using Mann-Whitney-test (α = 0.05). Corresponding *P*-values are shown.
(DOCX)

Table S4 **Medians of analyzed parameters in tip-recordings.**
(DOCX)

Materials and Methods S1 (DOCX)
