# Supplementary figures and images for: In situ Tip-Recordings Found No Evidence for an Orco-Based Ionotropic Mechanism of Pheromone-Transduction in Manduca sexta
Source: PLoS One. 2013 May 3;8(5):e62648. doi: 10.1371/journal.pone.0062648 (PMC3643954; doi:10.1371/journal.pone.0062648)

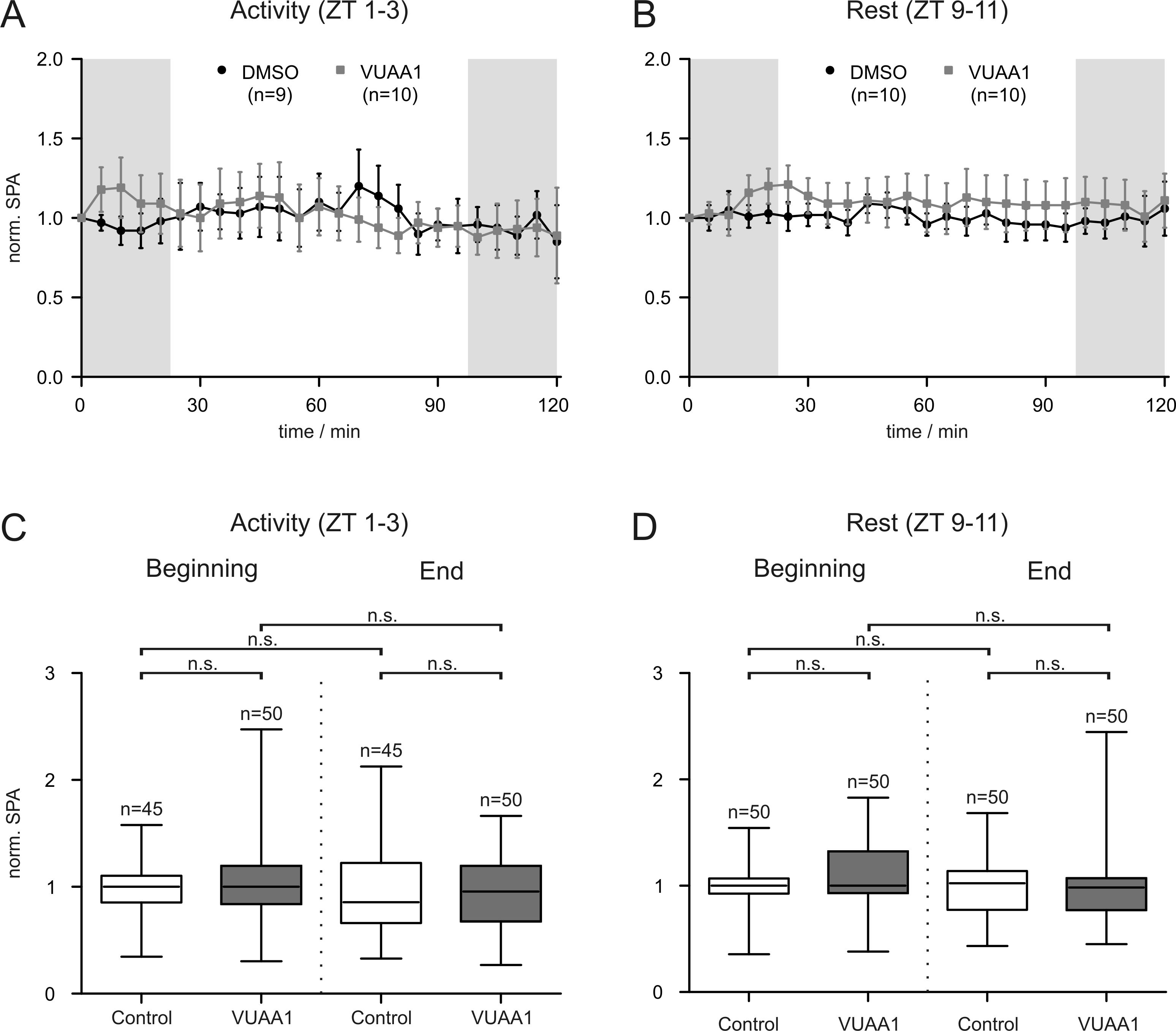

Supplement: Figure S1 — VUAA1 does not increase sensillum potential amplitude (SPA). (TIF) [file pone.0062648.s001.tif]

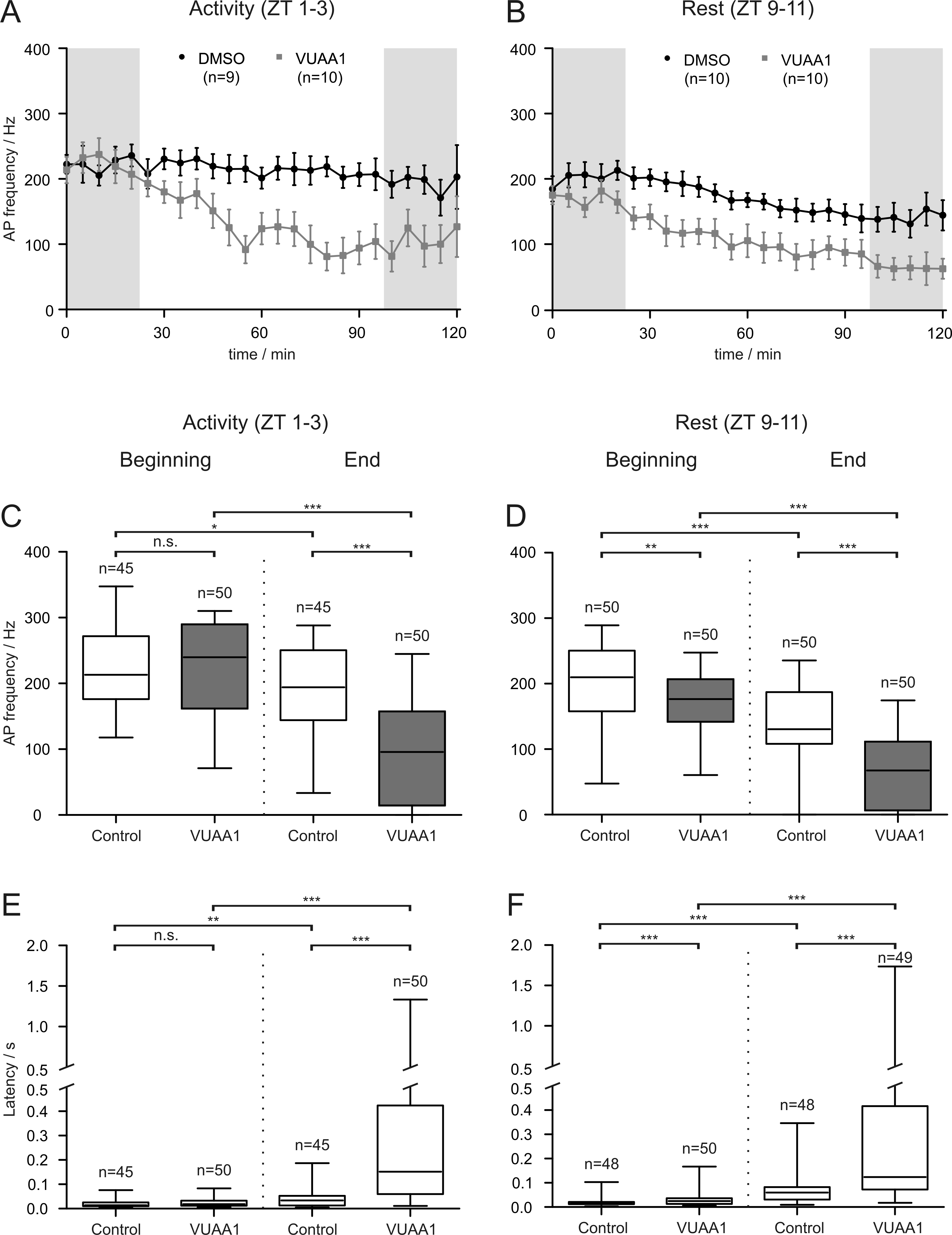

Supplement: Figure S2 — VUAA1-dependent MsexOrco activation affects the threshold of pheromone-responses during the course of the 2 h-long recording, except during the first 20 min at the activity phase. (TIF) [file pone.0062648.s002.tif]

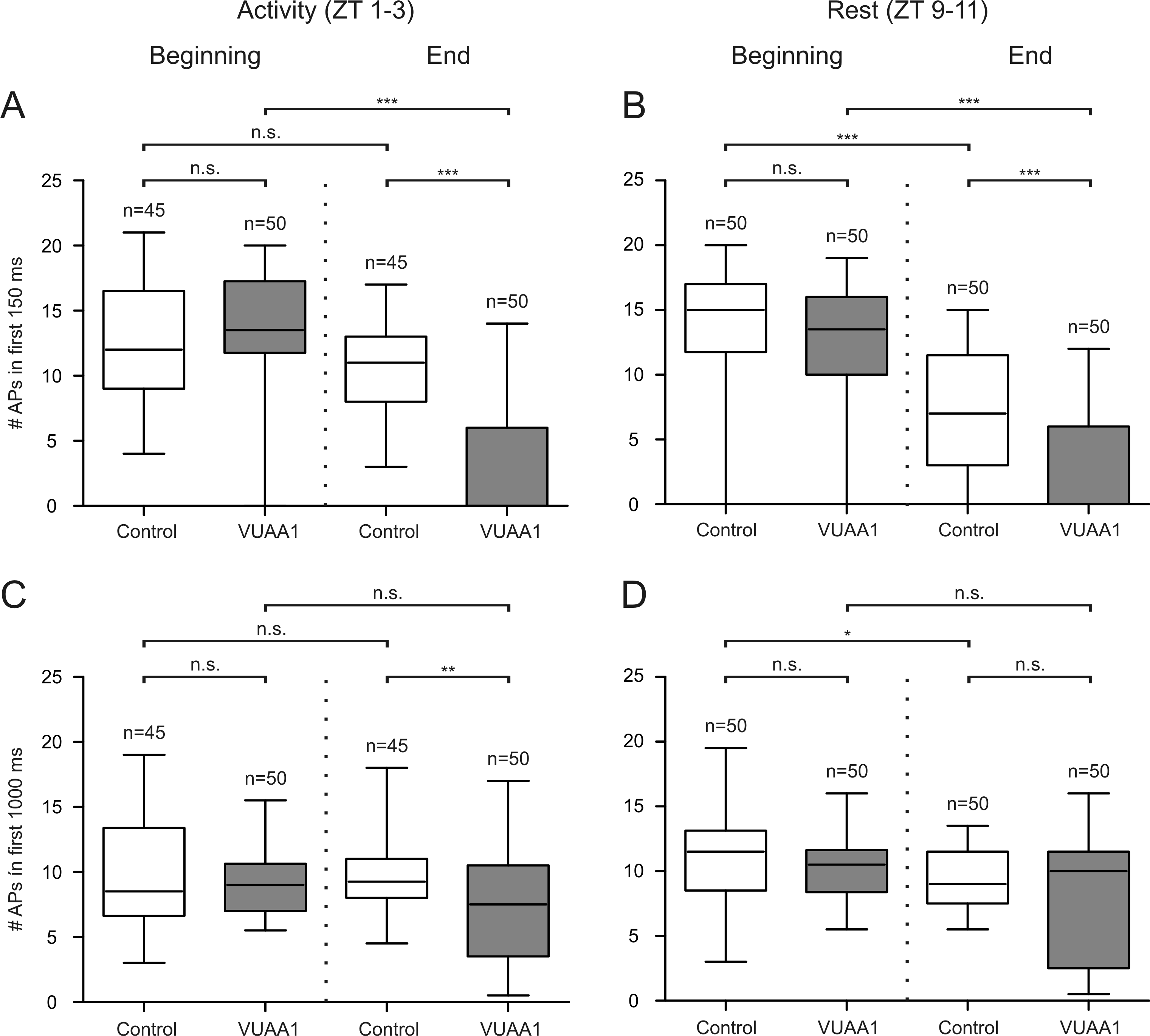

Supplement: Figure S3 — Orco agonist VUAA1 (100 µM) slows the kinetics of the bombykal (BAL) response. (TIF) [file pone.0062648.s003.tif]

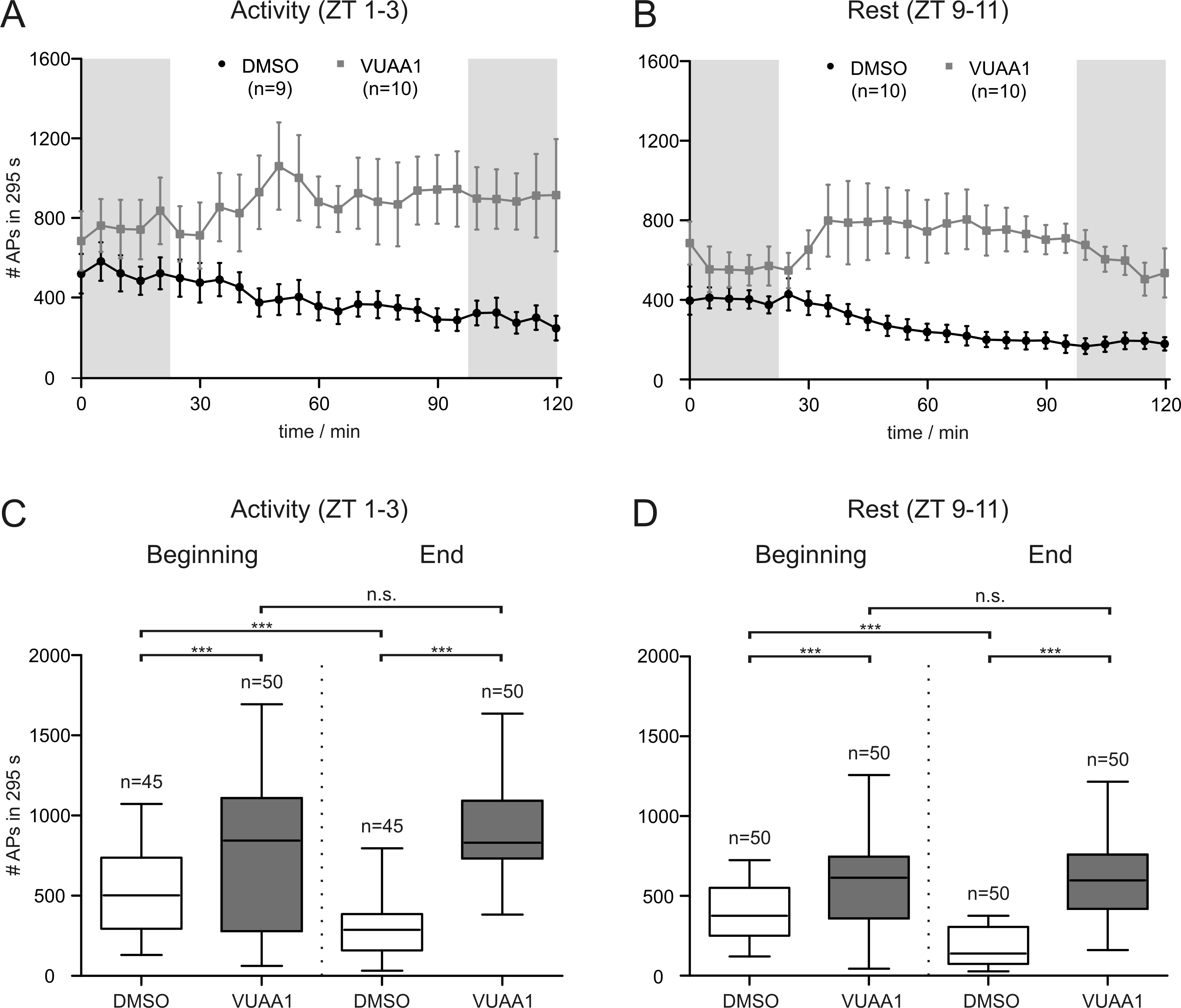

Supplement: Figure S4 — VUAA1 increased background activity over the time course of the 2 h-long recordings. (TIF) [file pone.0062648.s004.tif]
