# Supplementary material for: In situ Tip-Recordings Found No Evidence for an Orco-Based Ionotropic Mechanism of Pheromone-Transduction in Manduca sexta
Source: PLoS One. 2013 May 3;8(5):e62648. doi: 10.1371/journal.pone.0062648 (PMC3643954; doi:10.1371/journal.pone.0062648)
Supplement: Table S1 — Primer sequences. Coding sequences are shown in capitals. If a restriction site was induced, the respective sequence and appropriate enzyme is indicated with fat letters. Abbreviations: for = forward primer, rev = reward primer. (DOCX) [file pone.0062648.s005.docx]

| **Primer name** | **Primer sequence** |
| --- | --- |
| MsexOrco **BamHI** for | cgc **gga tcc** ATG ACC ATG CTT CTG CGG AA |
| MsexOrco **EcoRI** rev | ccg **gaa ttc** CTA TTT CAG CTG CAC CAA C |
| MsexOR-1 **KpnI** for | cc **ggt acc** ATG ATA TTT ATG GAC GAT CCT CTA TCA AAG |
| MsexOR-1 **XhoI** rev | ga **ctc gag** TTA GTT AGA AAC GGT GCG AAG AAA TG |
| MsexOR-4 **KpnI** for | cc **ggt acc** ATG AAG TTT TTT GTA GAC GGC AGC GAA ATA |
| MsexOR-4 **XhoI** rev | ga **ctc gag** TTA GCT CTC ATC TTT GGC GAT TGT TTG A |
| MsexSNMP-1 for | ATG CGG CTG GCA AGG GGA ATT AAG |
| MsexSNMP-1 rev | TTA CAT GTT GAT TTT TGG AGG CTC ATG AC |
