# Supplementary material for: In situ Tip-Recordings Found No Evidence for an Orco-Based Ionotropic Mechanism of Pheromone-Transduction in Manduca sexta
Source: PLoS One. 2013 May 3;8(5):e62648. doi: 10.1371/journal.pone.0062648 (PMC3643954; doi:10.1371/journal.pone.0062648)
Supplement: Table S2 — Statistics for tip-recordings. Data groups were compared using Mann-Whitney-test (α = 0.05). Corresponding P-values are shown. (DOCX) [file pone.0062648.s006.docx]

|  | **Data groups** | | **SPA** | **AP frequency** | **Latency** | **# APs 0-150** | **# APs 0-1000** | **Background activity** | **% of APs in bursts** | **# APs per burst** |
| --- | --- | --- | --- | --- | --- | --- | --- | --- | --- | --- |
| **begin vs. begin** | control ZT 1-3 | 1 µM VUAA ZT 1-3 | 0.855 | 0.572 | 0.191 | 0.898 | 0.124 | 0.040 | 0.001 | 0.044 |
|  | control ZT 1-3 | 100 µM VUAA ZT 1-3 | 0.340 | 0.694 | 0.052 | 0.146 | 0.484 | 0.012 | < 0.001 | 0.002 |
|  | 1 µM VUAA ZT 1-3 | 100 µM VUAA ZT 1-3 | 0.263 | 0.857 | 0.617 | 0.068 | 0.333 | 0.217 | 0.130 | 0.363 |
|  | control ZT 9-11 | 1 µM VUAA ZT 9-11 | 0.779 | 0.710 | 0.008 | 0.620 | 0.002 | < 0.001 | < 0.001 | 0.004 |
|  | control ZT 9-11 | 100 µM VUAA ZT 9-11 | 0.381 | 0.002 | 0.001 | 0.097 | 0.113 | 0.001 | 0.001 | 0.205 |
|  | 1 µM VUAA ZT 9-11 | 100 µM VUAA ZT 9-11 | 0.474 | 0.001 | 0.136 | 0.124 | 0.059 | 0.331 | 0.001 | 0.010 |
|  | control ZT 1-3 | control ZT 9-11 | 0.629 | 0.133 | 0.905 | 0.132 | 0.172 | 0.017 | 0.023 | 0.882 |
|  | 1 µM VUAA ZT 1-3 | 1 µM VUAA ZT 9-11 | 0.383 | 0.013 | 0.342 | 0.035 | 0.173 | 0.704 | 0.398 | 0.137 |
|  | 100 µM VUAA ZT 1-3 | 100 µM VUAA ZT 9-11 | 0.893 | 0.000 | 0.143 | 0.179 | 0.013 | 0.032 | < 0.001 | 0.005 |
| **begin vs. end** | control ZT 1-3 | control ZT 1-3 | 0.796 | 0.039 | 0.002 | 0.134 | 0.693 | < 0.001 | 0.693 | 0.014 |
|  | 100 µM VUAA ZT 1-3 | 100 µM VUAA ZT 1-3 | 0.194 | < 0.001 | < 0.001 | < 0.001 | 0.067 | 0.273 | 0.030 | < 0.001 |
|  | control ZT 9-11 | control ZT 9-11 | 0.904 | < 0.001 | < 0.001 | < 0.001 | 0.015 | < 0.001 | 0.000 | 0.050 |
|  | 100 µM VUAA ZT 9-11 | 100 µM VUAA ZT 9-11 | 0.145 | < 0.001 | < 0.001 | < 0.001 | 0.103 | 0.918 | < 0.001 | < 0.001 |
| **end vs. end** | control ZT 1-3 | 100 µM VUAA ZT 1-3 | 0.979 | < 0.001 | < 0.001 | < 0.001 | 0.007 | < 0.001 | < 0.001 | 0.387 |
|  | control ZT 9-11 | 100 µM VUAA ZT 9-11 | 0.657 | < 0.001 | < 0.001 | < 0.001 | 0.344 | < 0.001 | < 0.001 | 0.001 |
|  | control ZT 1-3 | control ZT 9-11 | 0.393 | < 0.001 | 0.000 | < 0.001 | 0.798 | 0.004 | 0.663 | 0.417 |
|  | 100 µM VUAA ZT 1-3 | 100 µM VUAA ZT 9-11 | 0.459 | 0.055 | 0.953 | 0.943 | 0.382 | < 0.001 | 0.348 | 0.387 |
