# Supplementary material for: In situ Tip-Recordings Found No Evidence for an Orco-Based Ionotropic Mechanism of Pheromone-Transduction in Manduca sexta
Source: PLoS One. 2013 May 3;8(5):e62648. doi: 10.1371/journal.pone.0062648 (PMC3643954; doi:10.1371/journal.pone.0062648)
Supplement: Table S3 — Statistics for tip-recordings: Spontaneous activity. Data groups were compared using Mann-Whitney-test (α = 0.05). Corresponding P-values are shown. (DOCX) [file pone.0062648.s007.docx]

| **Data groups** | | ***P*-Value** |
| --- | --- | --- |
| control ZT 1-3 | 1 µM VUAA ZT 1-3 | 0.001 |
| 1 µM VUAA ZT 1-3 | 10 µM VUAA ZT 1-3 | < 0.001 |
| 10 µM VUAA ZT 1-3 | 100 µM VUAA ZT 1-3 | < 0.001 |
| 100 µM VUAA ZT 1-3 | 500 µM VUAA ZT 1-3 | 0.075 |
| control ZT 9-11 | 1 µM VUAA ZT 9-11 | < 0.001 |
| 1 µM VUAA ZT 9-11 | 10 µM VUAA ZT 9-11 | 0.780 |
| 10 µM VUAA ZT 9-11 | 100 µM VUAA ZT 9-11 | < 0.001 |
| 100 µM VUAA ZT 9-11 | 500 µM VUAA ZT 9-11 | < 0.001 |
