# Supplementary material for: In situ Tip-Recordings Found No Evidence for an Orco-Based Ionotropic Mechanism of Pheromone-Transduction in Manduca sexta
Source: PLoS One. 2013 May 3;8(5):e62648. doi: 10.1371/journal.pone.0062648 (PMC3643954; doi:10.1371/journal.pone.0062648)
Supplement: Table S4 — Medians of analyzed parameters in tip-recordings. (DOCX) [file pone.0062648.s008.docx]

|  | **Data groups** | **norm. SPA** | **AP frequency / Hz** | **Latency / s** | **# APs 0-150** | **# APs 0-1000** | **Background activity / # APs in 295 s** | **% of APs in bursts** | **# APs per burst** | **Spont. Activity / # APs in 295 s** |
| --- | --- | --- | --- | --- | --- | --- | --- | --- | --- | --- |
| **Begin** | Control ZT 1-3 | 1.00 | 213.0 | 0.014 | 12.0 | 17.0 | 503.0 | 84.08 | 3.284 | 11 |
|  | 1 µM VUAA1 ZT 1-3 | 1.00 | 241.4 | 0.016 | 13.0 | 16.0 | 715.0 | 74.17 | 3.103 | 38 |
|  | 10 µM VUAA1 ZT 1-3 | - | - | - | - | - | - | - | - | 477.5 |
|  | 100 µM VUAA ZT 1-3 | 1.00 | 239.9 | 0.018 | 13.5 | 18.0 | 844.5 | 71.64 | 3.052 | 1178 |
|  | 500 µM VUAA ZT 1-3 | - | - | - | - | - | - | - | - | 894 |
|  | Control ZT 9-11 | 1.00 | 209.7 | 0.015 | 15.0 | 23.0 | 375.0 | 86.73 | 3.319 | 11 |
|  | 1 µM VUAA1 ZT 9-11 | 1.00 | 206.8 | 0.020 | 15.0 | 18.5 | 696.0 | 70.95 | 2.908 | 113 |
|  | 10 µM VUAA1 ZT 9-11 | - | - | - | - | - | - | - | - | 91 |
|  | 100 µM VUAA1 ZT 9-11 | 1.00 | 176.6 | 0.025 | 13.5 | 21.0 | 614.0 | 79.11 | 3.273 | 698 |
|  | 500 µM VUAA1 ZT 9-11 | - | - | - | - | - | - | - | - | 1083 |
| **End** | Control ZT 1-3 | 0.86 | 194.3 | 0.034 | 11.0 | 18.5 | 288.0 | 77.63 | 2.879 | - |
|  | 100 µM VUAA1 ZT 1-3 | 0.96 | 95.7 | 0.152 | 0.0 | 15.0 | 831.5 | 66.86 | 2.812 | **-** |
|  | Control ZT 9-11 | 1.02 | 130.4 | 0.060 | 7.0 | 18.0 | 139.5 | 79.60 | 3.034 | - |
|  | 100 µM VUAA1 ZT 9-11 | 0.98 | 67.5 | 0.124 | 0.0 | 20.0 | 598.0 | 65.74 | 2.785 | - |
