# Supplementary material for: In situ Tip-Recordings Found No Evidence for an Orco-Based Ionotropic Mechanism of Pheromone-Transduction in Manduca sexta
Source: PLoS One. 2013 May 3;8(5):e62648. doi: 10.1371/journal.pone.0062648 (PMC3643954; doi:10.1371/journal.pone.0062648)
Supplement: Materials and Methods S1 — (DOCX) [file pone.0062648.s009.docx]

**Supporting information**

**SI Materials and Methods**

**Animals**

Hatched larvae were raised on artificial diet [[modified after 1](#_ENREF_1)]. The adult stages were fed with a sugar solution offered in reaction vessels enclosed in pseudo-flowers made of filter paper treated with a synthetic blend of odors [[modified after 2](#_ENREF_2)]. All *M. sexta* stages were kept at 24-27 °C and 40-60 % relative humidity in a 17h:7h light:dark cycle. Male pupae were isolated and used for experiments 1-2 days after eclosion. Experimental animals were fixated in a teflon holder with one antenna fixed with dental wax (Boxing wax, Sybron/Kerr, Romulus, MI, USA).

**Odorant receptor expression in heterologous systems**

Antennal mRNA was reverse transcribed using the Superscript II Mix (Invitrogen, Carlsbad, CA, USA). The resulting cDNA was used as template to amplify respective coding sequences for MsexOrco, MsexOR-1, MsexOR-4 and MsexSNMP-1 via PCR, adding suitable restriction sites outside the coding sequence (for primers see **Tab. S1**). PCR products were cloned into pCR^®^II-TOPO^®^ vectors and transformed into
*E. coli* (One Shot® Top10 competent cells, Invitrogen). Plasmids were isolated (QIAprep Spin MiniPrep Kit) and treated with suitable restriction enzymes (NEB, Ipswich, MA). Fragments were gel-purified and subcloned into the pcDNA3.1(-) expression vector (Invitrogen). After further replication in *E. coli* the vector was isolated (QIAGEN Plasmid Maxi Kit) and stored at -20 °C.

**Solutions**

Sensillum lymph Ringer (in mM: KCl, 6.4; MgCl_2_, 12.0; CaCl_2_, 1.0; NaCl, 12.0; HEPES, 10.0; D-glucose, 354.0) and hemolymph Ringer (in mM: KCl, 171.9; MgCl_2_, 3.0; CaCl_2_, 1.0; NaCl, 25.0; HEPES, 10.0; D-glucose, 22.5) were adjusted to pH 6.5. The osmolarity was adjusted with mannitol to 475 mOsmol/l for sensillum lymph Ringer and 450 mOsmol/l for hemolymph Ringer. Ringer solution for HEK 293 cells (in mM: NaCl, 135; KCl, 5; MgCl_2_, 1; CaCl_2_, 1; HEPES, 10; D-glucose, 10) was adjusted to pH 7.4.

The Orco agonist VUAA1 [[3](#_ENREF_3)] N-(4-ethylphenyl)-2-((4-ethyl-5-(3-pyridinyl)-4H-1,2,4-triazol-3-yl)thio)acetamide was synthesized by the Research Group Mass Spectrometry/Proteomics (Max Planck Institute for Chemical Ecology, Jena, Germany). The substance was dissolved in dimethyl sulfoxide (DMSO) at a concentration of 100 mM and stored at -20°C. For experiments VUAA1 was diluted in the respective Ringer solution. VUAA1 working solution, bath ringer solution for calcium imaging as well as the control hemolymph Ringer solution contained 0.1 % DMSO.

**Stimulation with pheromone**

Charcoal-filtered and moistened air streamed into a Clampex software-controlled solenoid valve (PA 202 004 P, Staiger, Erligheim, Germany) directing the air into one of two air branches. One branch of the perfusion system supplied the recording site with a continuous stream of clean, humidified air. The second branch passed a valve-controlled glass cartridge with a filter paper loaded with 1 µg bombykal (E,Z-10,12-hexadecadienal; BAL) dissolved in 10 µl *n*-hexan (Merck, Frankfurt, Germany). The BAL was generously provided by Dr. T. Christensen (University of Tucson, AZ, USA) and Dr. J. Krieger (University of Hohenheim, Germany). Pheromone stimuli (50 ms) were delivered every 5 min to avoid adaptation or depletion of pheromone. Released pheromone was removed quickly by a vacuum system installed directly below the mounted moth. One tip-recording encompassed 24 stimulations and lasted for 2 h.

**Data Analysis**

Calcium Imaging: Regions of interest (ROIs) were defined with Tillvision software by marking the profile of each cell. If the number of cells was far higher than 100, the ROIs were selected randomly and limited to 100. For each ROI the average ratio was calculated. Mean change of the intracellular Ca^2+^ concentration (Δ[Ca^2+^]_i_) for the respective cells was determined from the area under the curve (AUC) over the time courses after normalization to [Ca^2+^]_i_ before VUAA1 application. Each AUC segment between two data points was calculated using the trapezoid method. AUC segments for the whole time course were summed and divided by the number of segments
(n-1) multiplied by the framesize (t_j+1_ - t_j_).

$$AUC=\frac{1}{2}\sum_{j=1}^{n-1} (([Ca^{2+}]_{i})_{j}+([Ca^{2+}]_{i})_{j+1})(t_{j+1}-t_{j})$$

$$mean\Delta([Ca^{2+}]_{i})=\frac{AUC}{(n-1)(t_{j+1}-t_{j})}$$

Further analysis was performed with Matlab (version R2012a, The MathWorks). First data (i.e. F_340_/F_380_-ratios for each cell) was normalized by calculating the mean of the first ten values of a measurement and the percentage deviation of the mean for each normalized value was calculated. Then the percentage of active cells for each experiment was determined. Cells were considered active, if the ratio exceeded the 20-fold standard deviation of the values measured before application. We discriminated between Orco positive cells (i.e. transfected with Msex-Orco and optionally cotransfected with a pheromone receptor candidate (MsexOR-1 or MsexOR-4) and/or MsexSNMP-1) and Orco negative cells (optionally transfected with MsexOR-1, MsexOR-4 and/or MsexSNMP-1, but not with Msex-Orco).

Tip-recordings: The electrophysiological response of the olfactory receptor neuron to a stimulus was recorded continuously for 5 s with a sampling rate of 20 kHz (Clampex 8, Molecular Devices, Sunnyvale, CA, USA). To analyze the sensillum potential amplitude the signal was low-pass filtered (cutoff frequency: 50 Hz; with median filter: time constant = 0.05 ms; smooth process: time constant= 0.01). For evaluating the AP frequency a high-pass filter with a cutoff frequency of 150 Hz was used.

Background activity between two stimuli and spontaneous activity without stimulation were recorded discontinuously for 295 seconds (Clampex 8, fixed length events of 12.75 ms) with a sampling rate of 19.6 kHz (Clampex 8, fixed length events) for each trigger event. As shown by Dolzer et al. [[4](#_ENREF_4)] in tip-recordings two types of APs could be distinguished according to their amplitude, with the larger amplitude cell being the BAL-sensitive cell and the smaller amplitude cell being sensitive for another component of the pheromone blend.

**References**

1. Bell RA, Joachim FG (1976) Techniques for rearing laboratory colonies of tobacco hornworms and pink bollworms. Ann entomol Coc Amer 69: 365-373.

2. Riffell JA, Lei H, Christensen TA, Hildebrand JG (2009) Characterization and coding of behaviorally significant odor mixtures. Curr Biol 19: 335-340.

3. Jones PL, Pask GM, Rinker DC, Zwiebel LJ (2011) Functional agonism of insect odorant receptor ion channels. Proc Natl Acad Sci U S A 108: 8821-8825.

4. Dolzer J, Krannich S, Fischer K, Stengl M (2001) Oscillations of the transepithelial potential of moth olfactory sensilla are influenced by octopamine and serotonin. J Exp Biol 204: 2781-2794.

**SI Figures and Tables**

**Figure S1.** **VUAA1 does not increase sensillum potential amplitude (SPA). (A,B)** The maximum SPA remained unchanged by VUAA1- dependent MsexOrco activation over the 2 h time course of recordings. **(C,D)** Comparison of the first and last 20 minutes (beginning and end; shaded areas in A,B) of the recordings revealed no significant change for the SPA in the presence of VUAA1 (100 µM) (n.s. = not significant, Mann-Whitney-Test).

**Figure S2.** **VUAA1-dependent MsexOrco activation affects the threshold of pheromone-responses during the course of the 2 h-long recording, except during the first 20 min at the activity phase. (A,B)** Time course of the AP frequency for recordings during activity and rest. Shaded areas show the first and last 20 minutes of the recordings (beginning and end). **(C,D)** VUAA1 infusion (100 µM) decreased bombykal (BAL)-dependent action potential frequency (first 6 APs) continuously during the 2 h recordings. **(E,F)** Also, the delay of the first AP of BAL responses increased with VUAA1. Significant differences are indicated by asterisks (n.s. = not significant; *P < 0.05, **P < 0.01, ***P < 0.001; Mann-Whitney test).

**Figure S3. Orco agonist VUAA1 (100 µM) slows the kinetics of the bombykal (BAL) response.** The BAL responses shift from a phasic to a more tonic response pattern. In the first 20 minutes (beginning) of all tip-recordings VUAA1 never affected BAL sensitivity during the first 150 ms **(A,B)** and 1000 ms **(C,D)**. In the last 20 minutes (end) of the tip-recordings the BAL responses shifted to a more tonic response pattern in the presence of VUAA1. Significant differences are indicated by asterisks (n.s. = not significant; *P < 0.05, **P < 0.01, ***P < 0.001; Mann-Whitney test).

**Figure S4. VUAA1 increased background activity over the time course of the 2 h-long recordings. (A,B)** Time course of the background activity for recordings during activity and rest. Shaded areas show beginning (first 20 minutes) and end (last 20 minutes) of the recordings. VUAA1-dependent MsexOrco activation affected background activity already within the first bin (295 s) of the recordings. **(C,D)** Decrease of the background activity in controls was counteracted by VUAA1. In the first as well as the last 20 minutes VUAA1 increased background activity significantly (n.s. = not significant; ***P < 0.001; Mann-Whitney test).

**Table S1. Primer sequences.** Coding sequences are shown in capitals. If a restriction site was induced, the respective sequence and appropriate enzyme is indicated with fat letters. Abbreviations: for=forward primer, rev=reward primer.

**Table S2. Statistics for tip-recordings.** Data groups were compared using Mann-Whitney-test (α=0.05). Corresponding *P*-values are shown.

**Table S3. Statistics for tip-recordings: Spontaneous activity.** Data groups were compared using Mann-Whitney-test (α=0.05). Corresponding *P*-values are shown.

**Table S4. Medians of analyzed parameters in tip-recordings.**
